# Diorganotin Complexes of a Thiosemicarbazone, Synthesis: Properties, X-Ray Crystal Structure, and Antiproliferative Activity of Diorganotin Complexes

**DOI:** 10.1155/2010/867195

**Published:** 2010-06-20

**Authors:** Joanna Wiecek, Dimitra Kovala-Demertzi, Zbigniew Ciunik, Maria Zervou, Mavroudis A. Demertzis

**Affiliations:** ^1^Sector of Inorganic and Analytical Chemistry, Department of Chemistry, University of Ioannina, 45110 Ioannina, Greece; ^2^Faculty of Chemistry, University of Wrocław, 14 F. Joliot-Curie Street, 50-383 Wrocław, Poland; ^3^Institute of Organic and Pharmaceutical Chemistry, National Hellenic Research Foundation, Vas. Constantinou 48, 11635 Athens, Greece

## Abstract

The synthesis and spectral characterization of novel diorganotin complexes with 3-hydroxypyridine-2-carbaldehyde thiosemicarbazone, H_2_L**(1)**, [SnMe_2_(L)] **(2)**, [SnBu_2_(L)] **(3)**, and [SnPh_2_(L)] **(4)** are reported. The single-crystal X-ray structure of complex [SnPh_2_(L)(DMSO)] **(5)** shows that the ligand is doubly deprotonated and is coordinated as tridentate ligand. The six coordination number is completed by two carbon atoms of phenyl groups. There are two similar monomers **5a** (Sn1) and **5b** (Sn51) in the asymmetric unit. The monomers **5a** and **5b** are linked through intermolecular hydrogen bonds of N–H–O and C–H–S type. C–H → *π*, intermolecular interactions, intra- and intermolecular hydrogen bonds stabilize this structure and leads to aggregation and a supramolecular assembly. The IR and NMR (^1^H, ^13^C and ^119^Sn) spectroscopic data of the complexes are reported. The in vitro cytotoxic activity has been evaluated against the cells of three human cancer cell lines: MCF-7 (human breast cancer cell line), T-24 (bladder cancer cell line), A-549 (nonsmall cell lung carcinoma) and a mouse L-929 (a fibroblast-like cell line cloned from strain L). Compounds **1**, **3**, and **4** were found active against all four cell lines. Selectivity was observed for complexes **3** and **4** which were found especially active against MCF-7 and T-24 cancer cell lines.

## 1. Introduction

Organotin(IV) compounds find wide applications as catalysts and stabilizers, and certain derivatives are used as biocides, as antifouling agents and for wood preservation. It has been observed that several diorganotin adducts show potential as antineoplastic and antituberculosis agents [1–4]. 

Thiosemicarbazone derivatives are of considerable interest due to their antibacterial, antimalarial, antiviral, and antitumor activitiy [5, 6]. In our laboratory, the chemistry of thiosemicarbazones has been an extremely active area of research, primarily because of the beneficial biological (namely, antiviral and antitumor) activities of their transition-metal complexes [7–9]. 3-Hydroxypyridine-2-carbaldehyde thiosemicarbazone (**1**) is a member of the so-called *α*-(*N*)-heterocyclic carbaldehyde thiosemicarbazones (HCTs), which are the most potent known inhibitors of ribonucleoside diphosphate reductase. Compounds (**1**) and 5-hydroxypyridine-2-carbaldehyde thiosemicarbazone have shown high anticancer activity in animal models but were found to be readily glucuronidated and rapidly excreted [10]. The multiple dissociation constants of the ligand **H_2_L (1)** and the crystal structure of he complex of Pd(II) with **1**, [Pd(HL)Cl] have been studied by us [11]. This work is an extension of previously studied complexes of thiosemicarbazones with palladium(II), platinum(II), zinc(II), and organotin(IV) with potentially interesting biological activities [12–14]. 

The present paper includes the interaction of SnPh_2_O (where R is methyl, butyl, and phenyl groups) with 3-Hydroxypyridine-2-carbaldehyde thiosemicarbazone (H_2_L) and the crystal structure of the complex [SnPh_2_(L)(DMSO)] (**5**). IR and NMR spectroscopic data are reported. The results of the cytotoxic activity of diorganotin complexes have been evaluated for antiproliferative activity in vitro against human cancer cell lines: MCF-7, T-24, A-549, and a mouse L-929.

## 2. Experimental

### 2.1. General and Instrumental

All reagents were commercially available (Aldrich or Merck) and used as supplied. Solvents were purified and dried according to standard procedures. Melting points (m.p.) were determined in open capillaries and are uncorrected. IR and far-IR spectra were recorded on a Perkin Elmer Spectrum GX Fourier transform spectrophotometer using KBr pellets (4000–400 cm^−1^) and nujol mulls dispersed between polyethylene disks (400–40 cm^−1^). The free ligand was dissolved in (CD_3_)_2_SO and ^1^H, ^1^H–^1^H COSY and ^13^C spectra were acquired on a BRUKER 300 MHz spectrometer. Compounds **2**–**4** were dissolved in CDCl3 and were spectroscopically characterized by the use of ^1^D and ^2^D NMR spectroscopy on a Varian 600 MHz spectrometer. Experimental data were processed using VNMR and WinNMR routines. Chemical shifts (*δ*) are reported in ppm while spectra were referenced by the standard experimental setup. ^119^Sn NMR spectra were acquired on the Varian 600 MHz and tin spectra were referenced by the use of external solution of Me_4_Sn in C_6_D_6_. The splitting of proton resonances in the reported ^1^H-NMR spectra is defined as s = singlet, d = doublet, t = triplet, and m = multiplet. The chemical shifts are reported in ppm for ^1^H and ^13^C NMR. Elemental analyses were carried out by the microanalytical service of the University of Ioannina, Greece.

### 2.2. Synthesis of the Ligand and the Complexes

**3-Hydroxypyridine-2-Carbaldehyde Thiosemicarbazone (1)**

The ligand was synthesized according to a published procedure [11]. The white powder was recrystallized from cold ethanol and was dried in vacuo over silica gel. Yield: 75%. Bright-yellow powder. M.p. 209°C. IR (cm^−1^): 3555 s, 3451 m *v*(OH); 3291 m, 3194 m, *v*(NH_2_, NH); 1639 s, *v*(C=N); 1229 s, *v*(C–O); 1098 s, *v*(NN); 827 s, *v*(C=S). ^1^H-NMR: 11.50 (s, OH); 9.62 (s, H–N(3); 8.12 (d, H–C(1), ^3^J = 4.2 Hz); 7.26 (dd, H–C(2) ^3^J = 8.5, ^4^J = 4.2 Hz); 7.30 (dd, H–C(3) ^3^J = 8.4, ^4^J = 1.4 Hz); 8.34 (s, H–C(6)); 8.00, 8.24 (br. s, NH2). ^13^C-NMR: 178.0 C(7); 153.1 C(4); 144.4 C(6); 141.1 C(1); 137.6 C(5); 125.3 C(2); 124.2 C(3). Anal. calc. for C_7_H_8_N_4_OS (196.04): C, 42.8; H, 4.1; N, 28.6; S, 16.3; found: C, 42.6; H, 3.9; N, 28.2; S, 16.0%.

**SnMe_2_(L) (2)**

Dimethyltin(IV) oxide (0.033 g, 0.2 mmole) and 3-hydroxypyridine-2-carbaldehyde Thiosemicarbazone (0.0392 g, 0.2 mmole) in benzene (20 mL) were stirred and were refluxed for 12 hours under azeotropic removal of water (Dean-Stark trap). The resulting clear solution was rotary evaporated under vacuum to a small volume (2 mL), chilled and triturated with diethyl ether to give a white solid. The powder was recrystallized from distilled diethyl ether and dried in vacuo over silica gel to give yellow solid; mp. 228–230°C, Yield 35%. IR (cm^−1^): 3296 m, *v*(NH_2_); 1580 s, *v*(C=N); 1176 s, *v*(C–O); 1111 s, *v*(NN); 804 w, *v*(C=S); 583 m, 564 *v*(Sn–C); 431 m, *v*(Sn–N); 231 m, *v*(Sn–O); 376s, **ν**(Sn–S). ^1^H-NMR: 8.09 (dd, H–C(1), ^3^J = 4.2,^ 4^J = 1.4 Hz); 7.18 (dd, H–C(2) ^3^J = 8.5, ^4^J = 4.2 Hz); 7.08 (d, H–C(3) ^3^J = 8.5,^ 4^J = 1.4 Hz); 8.76 (s, H–C(6)); 5.09 (br. s, NH_2_); 0.89 (s, C**H**_3_, ^2^J (^117/119^Sn–^1^H) = 35.7 Hz). ^13^C-NMR: 168.8 C(7); 163.4 C(4); 161.5 C(6); 140.2 C(1); 135.4 C(5); 127.6 C(2); 128.9 C(3); 6.22 (**C**H_3_, J (^117/119^Sn–^13^C) = 309 Hz). ^119^Sn NMR: *δ* = −94.5. Anal. calc. for C_9_H_12_N_4_OSSn (343.0): C, 31.5; H, 3.5; N, 16.3; S, 9.3; found: C, 31.5; H, 3.7; N, 16.0;, S, 9.1 %.

**SnBu_2_(L) (3)**

Dibutylltin(IV) oxide (0.0498 g, 0.2 mmole) and 3-hydroxypyridine-2-carbaldehyde Thiosemicarbazone (0.0392 g, 0.2 mmole) in benzene (20 mL) were stirred and were refluxed for 12 hours under azeotropic removal of water (Dean-Stark trap). The resulting clear solution was rotary evaporated under vacuum to a small volume (2 mL), chilled and triturated with diethyl ether to give a white solid. The powder was recrystallized from distilled diethyl ether and dried in vacuo over silica gel to give yellow solid; mp. 126–128°C, Yield 41%. IR (cm^−1^): 3292 m, *v*(NH_2_); 1577 s, *v*(C=N); 1173 s, *ν*(C–O); 1114 s, *v*(NN); 805 w, *ν*(C=S); 578 ms, 560 sh *ν*(Sn–C); 418 m, *ν*(Sn–N); 247 m, *ν*(Sn–O); 394 ms, *ν*(Sn–S). ^1^H-NMR: 8.05 (dd, H–C(1), ^3^J = 4.2, ^4^J = 1.3 Hz); 7.16 (dd, H–C(2) ^3^J = 8.5, ^4^J = 4.2 Hz); 7.07 (dd, H- C(3) ^3^J = 8.5, ^4^J = 1.3 Hz); 8.82 (s, H-C(6)); 5.22 (br. s, NH_2_); 0.87 (t, 7.3 Hz, H*δ*); 1.32 (m, H*γ*); 1.54, 1.64 (H*α*, *β*). ^13^C-NMR: 167.7 C(7); 162.5 C(4); 160.0 C(6); 138.7 C(1); 134.5 C(5); 126.8 C(2); 128.0 C(3); 26.9 (C*α*, J (^117/119^Sn−^13^C) = 265 Hz); 28.2 (C*β*); 27.3 (C*γ*); 14.6 (C*δ*). ^119^Sn NMR: *δ* = −194.4. Anal. calc. for C_16_H_18_N_4_OSSn (537.2): C, 42.2; H, 5.7; N, 13.1; S, 7.5; found: C, 42.0; H, 5.9; N, 13.2; S, 7.4 %.

**SnPh_2_(L) (4)**

Diphenylltin(IV) oxide (0.0578 g, 0.2 mmole) and 3-hydroxypyridine-2-carbaldehyde thiosemicarbazone (0.0392 g, 0.2 mmole) in benzene (20 mL) were stirred and were refluxed for 12 hours under azeotropic removal of water (Dean-Stark trap). The resulting clear solution was rotary evaporated under vacuum to a small volume (2 mL), chilled and triturated with diethyl ether to give a white solid. The powder was recrystallized from distilled diethyl ether and dried in vacuo over silica gel to give yellow solid : mp. 186–188°C, Yield 34%. IR (cm^−1^): 3269 m, *ν*(NH_2_); 1589 s, *ν*(C=N); 1185 s, *v*(C–O); 1118 s, *v*(NN); 808 m, v(C=S); 322 ms, 303 sh *ν*(Sn–C); 419 m, *ν*(Sn–N); 248 m, *ν*(Sn–O); 370 ms, *ν*(Sn–S). ^1^H-NMR: 8.02(d, H–C(1), ^3^J = 4.2 Hz); 7.18(dd, H-C(2), ^3^J = 8.5, ^4^J = 4.2 Hz); 7.29 (d, H-C(3), ^3^J = 8.5 Hz); 8.88 (s, H–C(6)); 5.37 (br. s, NH_2_); 7.82 (d, 7.7 Hz, Ho-Ph); 7.34–7.30 (m, Hm, p-Ph). ^13^C-NMR: 167.4 C(7); 164.0 C(4); 161.5 C(6); 140.2 C(1); 135.4 C(5); 127.8 C(2); 129.3 C(3); 142.1 (Sn-Cph, J (^117/119^Sn–^13^C) = 424 Hz); 135.8 (Co-Ph, ^2^J (^117/119^Sn–^13^C) = 28 Hz); 128.8 (Cm-Ph, ^3^J (^117/119^Sn–^13^C) = 37 Hz); 130.2 (Cp-Ph, ^4^J (^117/119^Sn–^13^C) = 8.7 Hz); ^119^Sn NMR: *δ* = −227.2. Anal. calc. for C_19_H_16_N_4_OSSn (663.1): C, 48.9; H, 3.5; N, 12.0; S, 6.9; found: C, 48.6; H, 3.5; N, 10.7; S, 6.8%.

### 2.3. X-Ray Crystallography

Crystals of complex **5**, suitable for X-ray analysis, were obtained by slow crystallization of **4 **from a mixture of solvents C_6_H_6_/toluene/DMSO/CH_3_CN. Crystal data **5** are given in Table 1, together with refinement details. All measurements of crystals were performed in low temperature using an Oxford Cryosystem device on a Kuma KM4CCD *κ*-axis diffractometer with graphite-monochromated Mo Ka radiation. The data were corrected for Lorentz and polarization effects. No absorption correction was applied. Data reduction and analysis were carried out with the Oxford Diffraction (Poland) Sp. z o.o (formerly Kuma Diffraction Wroclaw, Poland) programs. Crystal structure was solved by direct methods (program SHELXS97) and refined by the full-matrix least-squares method on all *F* ^2^ data using the SHELXL97 [15] programs. Nonhydrogen atoms were refined with anisotropic displacement parameters; hydrogen atoms were included from geometry of molecules and Δ*ρ* maps. During the refinement process they treated as riding atoms. Molecular graphics were performed from PLATON2003 [16, 17].

Crystallographic data, that is, atomic coordinates, thermal parameters, bond lengths, and bond angles (CCDC number 634270 for **5**), have been deposited with the Cambridge Crystallographic Data Centre. Copies of available material can be obtained, free of charge, on application to CCDC, 12 Union Road, Cambridge CB2 1EZ, UK, (fax: +44-1223-336033 or e-mail: deposit@ccdc.cam.ac.uk or http://www.ccdc.cam.ac.uk).

### 2.4. Antiproliferative Assay In Vitro

The results of cytotoxic activity in vitro are expressed as IC_50_-the concentration of compound (in *μ*M) that inhibits a proliferation rate of the tumor cells by 50% as compared to control untreated cells. The compounds **1**–**4 **were tested for their antiproliferative activity in vitro against the cells of four human cancer cell lines: against the cells of three human cancer cell lines: MCF-7 (human breast cancer cell line), T-24 (bladder cancer cell line), A-549 (nonsmall cell lung carcinoma), and a mouse fibroblast L-929 cell line. 

*Compounds.* Test solutions of the compounds tested (1 mg/mL) were prepared by dissolving the substance in 100 *μ*L of DMSO completed with 900 *μ*L of tissue culture medium. Afterwards, the tested compounds were diluted in culture medium to reach the final concentrations of 100, 50, 10, 1 and 0.1 ng/*μ*L. The solvent (DMSO) in the highest concentration used in test did not reveal any cytotoxic activity. *Cells.* The cell lines are maintained in the Cell Culture Collection of the University of Ioannina. Twenty-four hours before addition of the tested agents, the cells were plated in 96-well plates at a density of 10^4^ cells per well. The MCF-7 cells were cultured in the D-MEM (Modified Eagle's Medium) medium supplemented with 1% antibiotic and 10% fetal calf serum. L-929 cells were grown in Hepes-buffered RPMI 1640 medium supplemented with 10% fetal calf serum, penicillin (50 U/mL), and streptomycin (50 mg/mL). A-549 cells were grown in F-12K Ham's medium supplemented with 1% glutamine, 1% antibiotic/antimycotic, 2% NaHCO_3_, and 10% fetal calf serum. The cell cultures were maintained at 37°C in a humid atmosphere saturated with 5% CO_2_. Cell number was counted by the Trypan blue dye exclusion method. MCF-7, L-929, and A-549 cells were determined by the sulforhodamine B assay [18], while T-24 cells by the MTT assay [19]. The in vitro tests were performed as described previously [20].

## 3. Results and Discussion

### 3.1. Spectroscopy

#### 3.1.1. Infrared Spectroscopy

The bands at 3555 and 3451 cm^−1^ are assigned to *v*(OH) mode while the medium-strong intensity bands at around 3291 and 3194 cm^−1^ in the spectra of H_2_L are assigned to *v*(NH_2_) and *v*(NH), respectively. The significant changes in the ligand bands upon complexing are the decrease in *ν*(C=N) and increase in *ν*(N–N) and the absence of the large systematic shifts of *ν*_as_(NH_2_) to lower frequencies. These data indicate coordination through the nitrogen of the azomethine group and no interaction between the terminal amino nitrogen and the metal ion. The *ν*(CS) band at 827 cm^−1^is less intense in the complexes **2**–**4** and is shifted to a lower frequency, suggesting coordination of the metal through sulfur. Coordination of the thiolato S-atom was further indicated by a decrease in the energy of the thioamide band as well as by the presence of a band at ca. 370 cm^−1^ assignable to *ν*(Sn–S). An IR band at 1229 cm^−1^ for **1** was assigned to *ν*(C–O). This band was found to be shifted to 1185–1173 cm^−1^, in the spectra of the complexes **2**–**4**. Coordination of the phenolic-O atom was further indicated by the presence of a band at ca. 250–230 cm^−1^ assignable to *ν*(Sn–O). From the metal-ligand stretching vibrations, which are below 600 cm^−1^, it is possible to assign the bands characteristic for *ν*(SnC). Also, the bands at 394–370 cm^−1^and 431–418 cm^−1^ are assigned to *ν*(SnS) and *ν*(SnN), respectively, and the bands at 250–230 cm^−1^ are assigned at *ν*(SnO) stretching vibrations [20, 21].

#### 3.1.2. NMR Spectra

^1^H and ^13^C resonances of the ligand H_2_L as well as of the complexes **2–4** bearing di-methyl, n-butyl, and phenyl groups attached to the central tin atom were unambiguously assigned by the use of 2D ^1^H-^1^H gCOSY, ^1^H,  ^13^C-HSQC, and ^1^H,^13^C-HMBC experiments. 

In the ^1^H NMR spectra of (H_2_L) **(1)** the broad singlet at **δ** 11.60 was attributed to OH group in accordance with [22] and the broad signal at **δ** 9.75 ppm was assigned to NH group. These two groups apparently participate in H-bonding with the nucleophilic solvent molecules (DMSO) or with other ligand molecules. These two signals are abolished upon interaction with the metal, indicating deprotonation of these groups and possible coordination to the tin(IV) atom at **2–4**. The absence of peaks corresponding to the imino proton NH and OH proton in the spectrum of **2–4** indicates that the nitrogen and oxygen are present in the deprotonated form and the ligand is dideprotonated. A sharp resonance peak appearing at *ca.* 5 ppm in all complexes is attributed to the NH_2_ group. This is also corfirmed by integration of the ^1^H spectral profile while additionally the use of CDCl_3_ eliminates the formation of H-bonding or complexation with the participation of solvent molecules as was the case with the ligand alone. In the ^1^H-NMR spectra of the complexes **2–4**, the formyl H-atom H–C(6) was shifted upon coordination, which indicated variations in the electron density at position 6. This signal was shifted downfield, in accordance with a decreased electron density at site C6 in the complexes. 

The C=S resonance of the thiosemicarbazone moiety in the free ligand resonated at 178.0 ppm. All complexes showed an upfield shift of C7 peak in the order of ~10 ppm compared with the free ligand, indicating the complexation of tin(IV) to the sulphur atom which apparently is related with an increased electron density at this site on complexation, due probably to *π*-back bonding for thiolato sulphur [21]. All the complexes exhibit downfield shifts of the C3, C4, and C6. This deshielding, in accordance with decreased electron density upon complexation, is indicative of Sn–O and Sn–N (azomethine nitrogen) bonds. These data indicate that the complexes are formed by ligand deprotonation followed by metallation, a structural motive that seems to be stable both in the solid state and in CDCl_3_ solution. ^119^Sn chemical shifts of compounds **2**–**4** were found between −94.4 and −227.2 in accordance with five-coordinate tin center [23].

### 3.2. Molecular Structure

**Figure 1:**
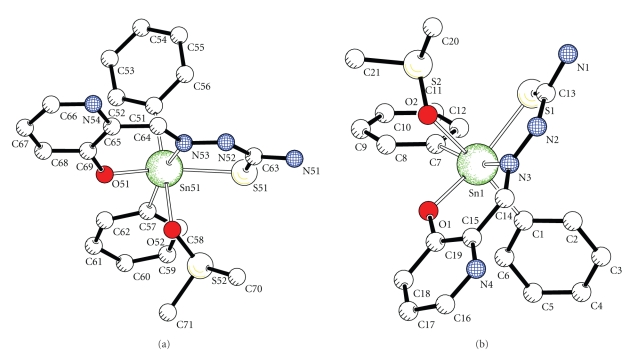
Molecular structure of the diorganotin complex **5**.

Crystals of complex **5**, suitable for X-ray analysis, were obtained by slow crystallization of **4 **from a mixture of solvents C_6_H_6_/toluene/DMSO/CH_3_CN. The crystal structure is shown in Figure 1. Crystal data are given in Table 1, together with refinement details. Bond lengths and angles are given in Table 2. There are two similar monomers **5a** (Sn1) and **5b** (Sn51) in the asymmetric unit. The double deprotonated ligand is coordinated as tridentate ligand *via* the phenolic oxygen O(1), the azomethine nitrogen N(3), and thiolato sulfur S(1) atoms. The molecule of DMSO is coordinated to the tin through oxygen O(2) atom. The six coordination number is completed by two carbon atoms of phenyl groups. The organic molecule acts as anionic tridentate with the ONS donors placed in the same side. The dianionic, tridentate ligand has a ZEZ configuration for the oxygen, nitrogen, and sulfur donor centers. The coordinated ligand consists of three rings, one heterocyclic and two chelates, SnSNNC and SnONCC, I and II, respectively. For monomers **5a **and **5b** the dihedral angles between the planes of the rings I and II are 14.47(6) and 12.59(6)°, respectively and between the ring II and the pyridyl ring are 7.4(5) and 8.1(2)°, respectively, indicating that the ligand as a whole in the two monomers deviates from planarity.

The C–S bond lengths 1.747(2), 1.749(2) Å for **5a** and **5b**, respectively, are shorter than a single bond (1.81 Å), but longer than a double bond (1.62 Å), suggesting partial single bond character. The C(14)–N(3) bond length, 1.301(2) Å, is close to a double bond (1.28 Å). The S–C bond distances are consistent with increased single-bond character while both thioamide C–N distances indicate increased double bond character. The negative charge of the deprotonated ligand is delocalized over the thiosemicarbazonato moiety. This is indicative of the coordinated thiosemicarbazone's greater conjugation and more delocalized electron density. The Sn–N(3) bond distance is longer than the sum of the covalent radii (2.15 Å), which indicates strong bond, while the bond distance Sn–S, 2.5141(5), 2.519(5) Å, though much shorter than the sum of the van der Waals radii (4.0 Å), indicates a weak bond [21]. The C–Sn C bond angle is equal to 105.87(7), 104.58(7)°, and the bond angles C(7)–Sn–N(3) and C(1) –Sn–O(2) are 160.47(6), 165.59(6) and 162.80(6), 166.89(6)° for **5a **and **5b**, respectively.

**Figure 2:**
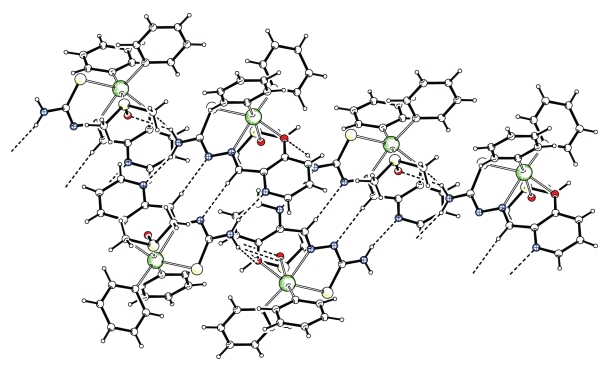
Arrangement of the intermolecular hydrogen bonds in **5**.

**Figure 3:**
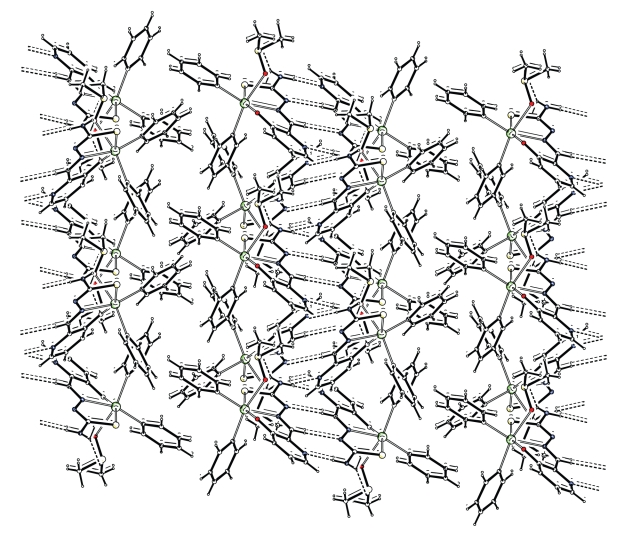
A view of the extended network of **5** along the *b *axis.

The two monomers **5a **and **5b **are linked through two intermolecular hydrogen bonds of N(51)–H(51B)–O(1) and of C(8)–H(8)–S(51) type (The N–O distance is 3.013(2) Å and the C–S distance is 3.673(2) Å. The monomers of **5a** form hydrogen-bonded dimers linked by two N(1)–H(1A)–N(4) hydrogen bonds involving the amino N(1)–H(1A) hydrogen atom and the pyridyl N(4) nitrogen. The monomers of **5a **are also linked by another C(14)–H(14)–N(2) hydrogen bond involving the formyl C(14)–H(14) hydrogen and the adjacent imino nitrogen N(2) and vice versa of centro-symmetrically related pairs of molecules. The observed hydrogen bonding patterns are of the DA=AD type. Also, the monomers of **5b** form hydrogen-bonded dimers and are linked by two hydrogen bonds, the N(51)–H(51A)–N(54) and C(64)–H(64)–N(52) (Figure 2). C–H→*π* intermolecular interactions intra- and intermolecular hydrogen bonds [24] stabilize this structure and lead to a supramolecular assembly, and Table 3 and Figure 3.

### 3.3. Pharmacology. Antiproliferative Activity In Vitro

Complexes of *N4*-ethyl 2-acetylpyridine thiosemicarbazone with platinum(II) or palladium(II) were tested in a panel of human tumor cell lines of different origins (breast, colon, and ovary cancers) and *cis*-*platin*-refractory/resistant cell lines and were found to exhibit very remarkable growth inhibitory activities with mean IC_50_ values of 0.9–0.5 nM and support the hypothesis that both [Pt(Ac4Et)_2_] and [Pd(Ac4Et)_2_] complexes can be characterized by cellular pharmacological properties distinctly different from those of* cis-platin* [8]. The complexes [ZnCl_2_(Fo4Npypipe)] and [ZnCl_2_(Ac4Npypipe)] where Fo4Npypipe and Ac4Npypipe are the monoion of 2-formyl pyridine N(4)-1-(2-pyridyl)-piperazinyl thiosemicarbazone and 2-acetyl pyridine N(4)-1-(2-pyridyl)-piperazinyl thiosemicarbazone have been evaluated in vitro against MCF-7, T-24, A-549, and L-929 cell lines and it was found to exhibit remarkable antiproliferative activity with mean IC_50_ values of 0.2–20 *μ*M [12]. The diphenylorganotin(IV) complex with pyruvic acid thiosemicarbazone has been tested against MCF-7, T-24, A-549, and L-929 cell lines and was found especially active against MCF-7 and T-24 cancer cells [21].

The antiproliferative activity of compounds is presented in Table 4 along with the activity of *cis-platin* and diorganotin(IV) oxides. Results showed that the ligand as well as the complexes **3** and **4 **demonstrated excellent antiproliferative activity, IC_50_ values range from 0.02–2.5 *μ*M, against all cell lines tested, while for *cis*-*platin* the IC_50_ values range from 0.7–41 *μ*M. The diorganotin(IV) oxides are nontoxic against L-929 and T-24 cancer cell lines and exhibit poor cytotoxic activity in A-549 and excellent antiproliferative activity against MCF-7 cancer cell line.

The ligand **1 **is more cytotoxic compared to *cis-platin* against the A-549, T-24 and MCF-7 cell lines and less cytotoxic against L-929 cell lines. The diorganotin complex **2 **is nontoxic against T-24 cell line, less cytotoxic against A-549 and L-929, and more cytotoxic against MCF-7 cell line compared to *cis-platin. *The diorganotin complexes **3 **and **4 **are in the same *μ*M range compared to *cis-platin *against L-929 and A-549 cancer cell lines and more cytotoxic against T-24 and MCF-7 cancer cell lines. The IC_50_ values for the ligand, **1**, against MCF-7 and T-24 cell lines are 0.15 *μ*M and 2.09 *μ*M, respectively, and against A-549 and L-929 cell lines are 0.81 *μ*M and 2.5 *μ*M, respectively. Ligand is 19.8 ± 1.3 and **52.4 **± 4.6 times more active than *cis-platin *against T-24 and MCF-7 cell lines, respectively. The IC_50_ values for **3** against MCF-7 and T-24 cell lines are 1.97 × 10^−2^ *μ*M and 1.1 *μ*M, respectively, and against A-549 and L-929 cell lines are 0.77 *μ*M and 1.05 *μ*M, respectively. Complex **3** is **403.6 **± 20.2 times more active than *cis-platin *against MCF-7 cell line and **37.9 **± 3.1 times more active than *cis-platin *against T-24 cell line. The IC_50_ values for **4** against MCF-7 and T-24 cell lines are 7.28 × 10^−2^ *μ*M and 0.73 *μ*M, respectively, and against A-549 and L-929 cell lines are 0.83 *μ*M and 1.37 *μ*M, respectively. Complex **4** is **110.3 **± 9.9 times more active than *cis-platin *against MCF-7 cell line and **57.1 **± 3.9 times more active than *cis-platin *against T-24 cell line. Compounds **1**, **3**, and **4** were found active against all four cell lines. Selectivity was observed for complexes **3 **and** 4** which were found especially active against MCF-7 and T-24 cancer cell lines. The mentioned evident differences in the antiproliferative action of the ligand and its diorganotin(VI) complexes indicate that these complexes really exist under the condition of the biological tests. Interestingly enough, **3 **and** 4 **were found to be more potent cytotoxic agent than the prevalent benchmark metallodrug, *cis-platin*, under the same experimental conditions measured by us. The superior activity of **3 **and** 4 **assumes significance in light of the fact that *cis*-*platin* is undisputedly the most studied and widely used metallopharmaceutical for cancer therapy known to date. It is noteworthy the high selectivity against MCF-7 and T-24 cancer cell lines.

## Figures and Tables

**Figure 4:**
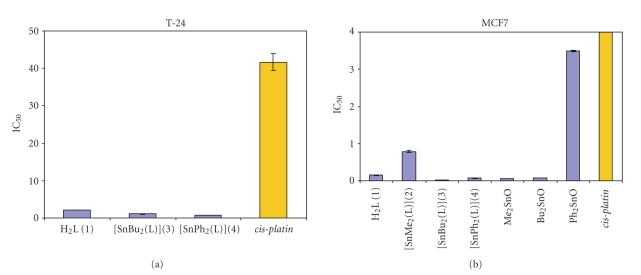
The antiproliferative activity in vitro expressed as IC_50 _± SD (*μ*M) against (a) T-24 and (b) MCF-7 cancer cell lines.

**Table 1:** X-ray crystal data and structure refinement.

	**5**
Empirical formula	C_21 _H_22_N_4_O_2_S_2_Sn
Formula weight	545.24
Temperature/ (K)	100 (2)
Wavelength/ (A)	0.71073
Crystal system	Triclinic
Space group	P-1
*a* (Å)	9.4663 (4)
*b* (Å)	14.7350 (7)
*c* (Å)	16.6374 (7)
*α* (°)	94.871 (4)
*β* (°)	96.434 (4)
*γ* (°)	90.793 (4)
Volume (A^3^)	2297.1 (2)
*Z*	4
Dc (Mg/m^3^)	1.577
Absorption coefficient (mm^−1^)	1.319
F(000)	1096
Crystal size (mm)	0.32 × 0.28 × 0.16
Diffractometer	Kuma KM4CCD
Theta range for data collection (°)	3.14–36.65
Ranges of *h, k, l *	−15→15, −24→20, −27→27
Reflections collected	35235
Independent reflections (Rint)	18670 (0.0381)
Completeness to 2*θ* = 36.65	81.9%
Data/parameters	18670/541
Goodness-of-fit (*F* ^2^)	0.920
Final R1/wR2 indices [I>2*σ*(I)]	0.0336/0.0768
Largest diff. peak/hole (e/Å^3^)	2.090/−1.098

**Table 2:** Bond lengths (Å) and angles (°) for complex 5.

**5a**		**5b**	
Sn(1)–S(1)	2.5141(5)	Sn(51)–S(51)	2.5190(5)
Sn(1)–O(1)	2.087(2)	Sn(51)–O(51)	2.088(2)
Sn(1)–O(2)	2.337(2)	Sn(51)–O(52)	2.345(2)
Sn(1)–N(3)	2.251(2)	Sn(51)–N(53)	2.262(2)
Sn(1)–C(1)	2.164(2)	Sn(51)–C(51)	2.157(2)
Sn(1)–C(7)	2.149(2)	Sn(51)–C(57)	2.151(2)
S(1)–C(13)	1.747(2)	S(51)–C(63)	1.749(2)
S(2)–O(2)	1.528(2)	S(52)–O(52)	1.528(2)
S(2)–C(20)	1.791(3)	S(52)–C(70)	1.777(3)
S(2)–C(21)	1.789(2)	S(52)–C(71)	1.787(3)
O(1)–C(19)	1.326(2)	O(51)–C(69)	1.320(2)
N(2)–N(3)	1.380(2)	N(52)–N(53)	1.375(2)

S(1)–Sn(1)–O(1)	156.25(4)	S(51)–Sn(51)–O(51)	155.68(4)
S(1)–Sn(1)–O(2)	84.87(3)	S(51)–Sn(51)–O(52)	83.23(3)
S(1)–Sn(1)–N(3)	77.37(4)	S(51)–Sn(51)–N(53)	77.71(4)
S(1)–Sn(1)–C(1)	101.71(5)	S(51)–Sn(51)–C(51)	100.07(5)
S(1)–Sn(1)–C(7)	95.51(5)	S(51)–Sn(51)–C(57)	100.30(5)
O(1)–Sn(1)–O(2)	76.08(5)	O(51)–Sn(51)–O(52)	77.25(5)
O(1)–Sn(1)–N(3)	84.11(5)	O(51)–Sn(51)–N(53)	83.46(5)
O(1)–Sn(1)–C(1)	94.05(6)	O(51)–Sn(51)–C(51)	95.98(6)
O(1)–Sn(1)–C(7)	97.14(6)	O(51)–Sn(51)–C(57)	93.14(6)
O(2)–Sn(1)–N(3)	75.34(5)	O(52)–Sn(51)–N(53)	75.65(5)
O(2)–Sn(1)–C(1)	165.59(6)	O(52)–Sn(51)–C(51)	166.89(6)
O(2)–Sn(1)–C(7)	86.01(6)	O(52)–Sn(51)–C(57)	87.15(6)
N(3)–Sn(1)–C(1)	93.44(6)	N(53)–Sn(51)–C(51)	92.55(7)
N(3)–Sn(1)–C(7)	160.47(6)	N(53)–Sn(51)–C(57)	162.80(6)
C(1)–Sn(1)–C(7)	105.87(7)	C(51)–Sn(51)–C(57)	104.58(7)

**Table 3:** C–H→*π* interactions and intermolecular hydrogen bonds for 5.

C–H(I) → Cg(J)^a^			H–Cg	C–Cg	∠C–H–Cg

C(11)–H(11) [1] → Cg(4)^(i)^			2.71	3.5394	159
C(60)–H(60) [2] → Cg(5)^(ii)^			2.66	3.4118	135

D	H	A^b^	H ⋯ A	D ⋯ A	∠D–H ⋯ A

N(1)–H(1A)⋯N(4)^(iii)^			2.13	3.005(2)	165
N(1)–H(1B)⋯O(51)^(iv)^			2.28	3.065(2)	150
N(1)–H(1B)⋯O(52)^(iv)^			2.55	3.124(2)	124
N(51)–H(51A)⋯N(54)^(v)^			2.16	2.993(3)	168
N(51)–H(51B)⋯O(1)			2.24	3.013(2)	152
C(8)–H(8)⋯S(51)			2.84	3.673(2)	143
C(14)–H(14)⋯N(2)^(iii)^			2.50	3.442(2)	175
C(58)–H(58)⋯S(51)			2.85	3.564(2)	127
C(62)–H(62)⋯O(51)			2.55	3.132(2)	118
C(64)–H(64)⋯N(52)^(v)^			2.58	3.483(2)	174

^a^Where Cg(4) and Cg(5) are referred to the rings C(1)–C(6) and C(7)–C(12); ^b^Cg–Cg is the distance between ring centroids; symmetry transformations, (i) 1 − *x*, −*y*, −*z*; (ii) 1−*x*, 1−*y*, −*z*; (iii) 1−*x*, −*y*,1−*z*; (iv) *x*, −1 + *y*, *z*; (v) 2 − *x*, 1 − *y*, 1 − *z*.

**Table 4:** The antiproliferative activity in vitro of 1–4, expressed as as IC_50_ ± SD (*μ*M) against MCF-7, T-24, A-549, and L-929 cancer cell lines.

	L929	A549	T24	MCF7
**H_2_L (1)**	2.5 ± 0.03	**0.81± 0.02**	2.09 ± 0.03	**0.153 **±** 0.01 **
[SnMe_2_(L)] (**2**)	7.29 ± 0.04	9.04 ± 0.0*5 *	<292	**0.79 **±** 0.03**
[SnBu_2_(L)] (**3**)	1.05 ± 0.02	**0.77 ± 0.03**	1.1 ± 0.05	1.97 × 10^−2^ ± 0.2 × 10^−2^
[SnPh_2_(L)] (**4**)	1.37 ± 0.03	**0.83 ± 0.02**	**0.73 **±** 0.02**	7.28 × 10^−2^ ± 0.5 × 10^−2^

Me_2_SnO	<607	17.9 ± 0.86	<607	6.0 × 10^−2^ ± 0.2 × 10^−2^
Bu_2_SnO	<402	10.4 ± 0.41	<402	8.1 × 10^−2^ ± 0.4 × 10^−2^
Ph_2_SnO	10.7 ± 0.5	47.1 ± 0.49	<346	3.5 ± 0.02

**cisplatin**	0.69 ± 0.03	1.53 ± 0.10	41.66 ± 2.2	7.99 ± 0.31
